# Antiseptic Dressing

**Published:** 1890-09

**Authors:** G. M. Pease

**Affiliations:** San Francisco, Cal.


### ANTISEPTIC DRESSING.

G. M. PEASE, M. D., SAN FRANCISCO, CAL.

Having recently witnessed what I suppose is called antiseptic dressing after a surgical operation, the absurdity of it as a protective against the incursions of the deadly (!) microbe forced itself upon me, and the peculiar trait of the ostrich which thinks to elude its pursuers by hiding its head seems on a par with gauze and tissue dressing.

Let us suppose an abdominal section has been completed, the tissues nicely stitched, edges accurately coaptated. Now, a few thicknesses of iodoform gauze, over this some bichloride gauze, over this a sheet of gutta-percha tissue, then some other absorbent and more gutta percha, or perhaps only a bandage to hold the rest in place.

Who will say there is not enough in the way to prevent even the most daring and energetic microbe from effecting an entrance to the well-covered wound. Like the vulture of the desert the microbe scents the distant prey, but first he must work his way through the bandage. When he has taken sufficient rest from this labor he leisurely treads the labyrinth of absorbent down to the gutta percha. Ah! here is a tough bit for him to get through. He is tired, but still hungry for the coveted wound. Perhaps he gets through the gutta percha, but alas, only to find a deadly poison in the mercurial protective, but bravely he goes forward, preferring death to defeat, until he meets the iodoform. Nearly dead he meets this new poison, which proves too much for him, and he quietly gives up the ghost and the wound is spared the presence of its deadly foe. Great and wonderful is this discovery for the protection of all surgical wounds !

Now we have supposed the microbe to have attacked this dressing directly from the front. If he has any cunning he will not act in that way, but make a flank movement and approach from a little distance following the skin and quietly crawl *under* the bandage, meeting no opposition until he perceives the odor of iodoform.

But as iodoform has been shown to possess little or no germicidal power he cares nothing for its being over his head and he is free to carry out his mission as a scavenger of unhealthy matter.

Perhaps it has been thought advisable to leave a drainage-tube in the abdomen ; what greater inducement could be offered than this open highway for the approach of his germic majesty not only to the external wound but even into the innermost recesses of the abdominal cavity.

Human nature is so constituted in many instances as to require a great deal of pomp and fuss in order to convey one little fact to the brain, and in this case the little lesson is cleanliness. It does not require a massive frame about a gem of a picture to make the picture appreciated by an artist ; he only regards the picture and laughs at the absurdity of its framing.

Nature has provided an excellent seal in the serum of the blood. If circumstances are such that this serum will not be properly thrown out, and it is necessary to protect a wound from the air, why not imitate nature's sealing ; and, in what way can it be better done than by applying collodion. The deadly (!) microbe cannot, at least, crawl under this. Other advantages exist besides the air-tight sealing : because transparent it allows the frequent observation of what is going on under it, without the labor of removal and replacing of a pile of gauzes and tissues ; it holds the skin firmly and assists the sutures in their work ; in contains no poisons for possible absorbance by the system.

One word, however, in favor of the antiseptic dressing. It is a revolution against the old-fashioned wet dressing, and in favor of dry dressing, and, perhaps, it requires all the chemicals and gauzes and paraphernalia to impress upon the average surgeon the value of a dry dressing. For that reason, and that only, is it of value.

Observation will probably show that all, or nearly all, of those who are great sticklers for antiseptics in surgery are wedded to the hypodermic syringe, and use it upon the slightest provocation. If a scalpel or forceps or other instrument must be soaked in antiseptic fluids, and such great care taken against the frisky microbe, why are they so careless with the syringe?

The same tube does duty in persons with all sorts of diseases, and it is safe to say that usually that tube is never more than carelessly wiped before it is put back into its case.

What a fine hiding place for sepsis is that tube and its surroundings.

"Consistency, thou art a jewel."

